# A multi-center trial-based economic evaluation of the SELF-program: A function-focused care program for nursing staff providing long-term care for geriatric clients in nursing homes compared to care as usual

**DOI:** 10.1371/journal.pone.0320649

**Published:** 2025-07-02

**Authors:** Ruben M.W.A. Drost, Ghislaine A.P.G. van Mastrigt, Stan Vluggen, Silke F. Metzelthin, Michel H.C. Bleijlevens, Getty Huisman-de Waal, Janneke M. de Man-van Ginkel, Sandra M.G. Zwakhalen

**Affiliations:** 1 Department of Health Services Research, Maastricht University, Care and Public Health Research Institute, Maastricht, The Netherlands; 2 Maastricht Health Economics and Technology Assessment Centre, Maastricht, The Netherlands; 3 Living Lab in Ageing and Long-Term Care, Maastricht, The Netherlands; 4 Zuyd University of Applied Sciences, Heerlen, The Netherlands; 5 Radboud University Medical Center, Nijmegen, The Netherlands; 6 Leiden University Medical Center, Leiden, The Netherlands; Wingate University, UNITED STATES OF AMERICA

## Abstract

**Introduction:**

This study aims to evaluate the cost-effectiveness and cost-utility of the SElf-reliance, autonomy, Life quality, and Functionality-program (SELF) for nursing staff in nursing homes who provide long-term care to geriatric clients as compared to care as usual from a societal perspective.

**Methods:**

The economic evaluation ran parallel to a two-arm multicenter cluster-randomized trial, in which the nursing staff in the intervention group received the SELF-program, and nursing staff in the control group received no program and delivered care as usual. Outcomes and societal costs for clients who received care from nursing staff were measured using questionnaires at baseline, 3 months, and 6 months. The main measures included Activities of Daily Living, as measured with the GARS-4, utility scores as measured with the EQ-5D-5L, and costs related to the intervention, informal care and health service utilization. Cost-effectiveness and cost-utility analyses were conducted, calculating incremental cost-effectiveness ratios from societal and healthcare perspectives. Bootstrap analyses were performed, with results displayed on cost-effectiveness planes and acceptability curves.

**Results:**

In total, 28 wards with a total of 241 clients were randomized (intervention, n = 115; control group, n = 126) From a societal perspective, care delivered in the intervention group led to lower costs than care as usual over the 6-month period, with incremental costs of -€584. In terms of the GARS-4 sum score, the SELF-program resulted in a favorable decrease of 0.81 points, and in terms of QALYs, it resulted in a favorable increase of 0.07. From a healthcare perspective, the incremental costs amounted to €556, with ratios of €410 per point reduction on the GARS-4 and €8,356 per QALY.

**Conclusions:**

The main analysis suggests that over 6 months from a societal perspective for both outcomes, the intervention is cost-effective as compared to care as usual.

**Trial registration:**

International Clinical Trials Registry Platform: NL9189; https://trialsearch.who.int/Trial2.aspx?TrialID=NL9189

## Introduction

Given their intensive contact to those in need of care, nursing staff plays a crucial role in encouraging and enabling nursing home clients to maximize their level of activity, functional ability, independence, and quality of life [1]. Although nursing staff play a crucial role, they often tend to work in a task-oriented manner and, although well-intentioned, unnecessarily take over tasks from clients [2,3]. Several studies have demonstrated that such actions deprive nursing home clients of their remaining abilities, compromise their dignity and quality of life, and may ultimately lead to disability [4–6]. From a nursing perspective, unnecessarily taking over tasks may result in unwarranted care consumption and higher workload. High nursing workload is well known to have a negative impact on nursing staff and client outcomes [7]. Moreover, from an economic perspective, this task-oriented working approach could lead to unnecessary care provision, potentially accelerating health deterioration and, in turn, resulting in increased healthcare costs.

Care approaches like Function-Focused Care (FFC), which is similar to reablement [8], try to change the care philosophy from doing things for the clients towards doing things with the client. This means that day-to-day services are meant to be goal-oriented, holistic and person-centered, considering the strengths and capabilities of clients instead of focusing on disease and dependency [9]. By enhancing clients’ strengths and capabilities, FFC has the potential to improve clients’ health-related quality of life and reduce the need for care resources [10]. This makes FFC a promising intervention from a societal perspective.

In practice, available FFC-based and reablement interventions have shown themselves feasible but have yielded mixed results in improving care professionals’ activity encouragement behavior and clients’ engagement in functional and physical activity [11,12]. In the Netherlands, various interventions with a similar basis have been developed, implemented, and evaluated in inpatient and outpatient long-term care settings [13–16]. Consistent with internationally developed interventions [17], these Dutch programs have produced mixed results about client outcomes. Moreover, the number of studies assessing the cost-effectiveness of such interventions is limited. Only studies assessing outpatient care interventions are available, and they have shown mixed results [18–20]. Specifically, a reablement training program for homecare staff was found to be not cost-effective compared to care as usual [20]. Until now no economic evaluations have been performed of inpatient FFC programs..

Based on the lessons learned from the abovementioned prior studies in this field, a list of five principles has been compiled to further optimize FFC-based programs and enhance their chances of effectiveness and cost-effectiveness [1,11,21]. These principles include: 1) tailoring the program to specific elements and needs of its participants, 2) addressing FFC components jointly rather than focusing on individual elements, 3) including an interactive training component that encourages participant ownership, 4) incorporating an extended integrated theory of behavior change, and 5) Improving managerial support by ensuring adequate time and staff resources. These principles have been integrated into the “SElf-reliance, autonomy, Life quality, and Functionality” (SELF) program, a training program designed for nursing staff providing long-term care for geriatric clients [1]. The objective of this economic evaluation is to assess the cost-effectiveness and cost-utility of the SELF-program for nursing staff in nursing homes who provide long-term care to geriatric clients as compared to care as usual from a societal perspective.

## Methods

### Design

The economic evaluation was conducted alongside a two-arm multicenter cluster-randomized trial (CRT) comparing the SELF-program to care as usual (CAU). The trial was registered in the Dutch Trial Register (NL9189), which has been adopted in the International Clinical Trial Registry Platform as of 2022. Fourteen nursing home wards were randomized to the intervention condition, and fourteen were assigned to the control condition following the randomization procedure presented in the protocol paper of the SELF-program [1]. Data collection and analysis followed the Dutch guidelines for economic evaluations in healthcare [22]. Following these guidelines, a societal perspective was adopted, considering both healthcare and non-healthcare costs. In addition, an analysis was conducted from a healthcare perspective, excluding informal care costs. Both a cost-effectiveness analysis (CEA) and a cost-utility analysis (CUA) were performed. A six-month follow-up was chosen because of the advanced age of the nursing home clients and their limited length of stay there. In the CEA and CUA, incremental costs were compared with the incremental effects of the treatments examined in the trial (SELF-program versus care as usual). The reporting adheres to the CHEERS 2022 guideline [23]. The CHEERS statement can be found in the supporting information (S1 Table).

### Intervention

The intervention comprised a training program for nursing staff including seven face-to-face sessions, ranging from one to two hours each, conducted over three months. Each session included interactive assignments aimed at promoting bottom-up learning and participant autonomy. The sessions were facilitated by a trainer selected from the organization, typically an individual with a background in education or quality improvement. Furthermore, one of the participating nursing staff members volunteered as an assistant trainer to provide relevant examples from daily care practice and support the main trainer during the sessions. The training program covered the essential elements of the FFC philosophy, with a particular emphasis on fostering behavior change in nursing staff to enhance activity encouragement [24].

The training program commenced with a two-hour kickoff session aimed at raising awareness about activity encouragement behavior among nursing staff. It also addressed and promoted the organizational policy concerning the self-reliance and independence of clients. In the following sessions, various themes were covered:

1. Session 1: Fostering awareness, exploring attitudes, and emphasizing the need for behavior change to enhance activity encouragement behavior.
2. Session 2: Motivation and willingness to change behavior, along with the importance of consistent application of the desired behavior among the care team.
3. Session 3: Translating willingness into concrete goals and actions to facilitate actual behavior change.
4. Booster Session 1: Reviewing the environment where geriatric clients reside.
5. Session 4: Conducting an actor-guided role-play session, involving an external expert in healthcare, to practice activity encouragement behavior in a real-life and interactive manner.
6. Session 5: Emphasizing the importance of continued motivation and mentoring for both nursing staff and clients to sustain the changed behavior.
7. Booster Session 2: Recapitulating the content of previous sessions to ensure the nursing care team is adequately prepared for reinforcing behavior maintenance and self-regulation.

The full outline of the intervention and its theoretical underpinnings are discussed elsewhere [1]. The control condition received CAU, which in the context of this study means that nursing staff allocated to usual care did not receive the training and provided care as usual to their clients.

### Recruitment of participants

Participants were recruited from nursing homes belonging to three large geriatric care organizations located in the southern and central regions of the Netherlands. Recruitment took place from March 2nd, 2021, to July 8th, 2022. All nursing staff members who were part of a nursing home ward were eligible to participate in the training. Those who were not involved in care delivery in activities of daily living (ADL) were excluded. Geriatric clients were eligible to participate if they were residing at the concerned ward at time of ward inclusion. Clients were excluded from participation when aged < 60 years old, or when terminally ill or bed-bound. All nursing staff and clients allocated to either the intervention or control condition received an information letter and an informed consent form before the start of the study. Informed consent could be provided by either the client or their primary contact person [1].

### Data collection

Data for the economic evaluation of the SELF-program were measured at the client level and collected from the primary contact person within the nursing team, either with or without direct involvement of the client. To address uniformity in data-collection, all client data were collected by the primary responsible caregiver, independent of the client’s somatic or psychogeriatric background. Similar to the effect evaluation, digitalized questionnaires were utilized using Qualtrics as the data collection platform [25]. Collected background information included age, gender (male, female, or open answer option), education level (low; no education up to lower technical education, medium; general secondary education up to secondary vocational education or high; school of higher general secondary education up till university degree), marital status (married, in a relationship, single/divorced, widow(er)), and care duration in nursing home. Information regarding client resource utilization was collected at the same time points as the health outcomes, namely at baseline (T0), directly after completion of the SELF-program for nursing care professionals at 3 months (T1), and 6 months (T2).

### Resource use and costing

All unit prices obtained from the National guidelines and associated costs were adjusted for 2022 prices using indices provided by Statistics Netherlands [26]. As the follow-up period was less than a year, discounting was not applied. Supporting information (S2 Table) contains the unit prices used for calculating intervention and healthcare costs.

#### Intervention costs.

The primary intervention costs encompassed expenses associated with personnel training, which involved time commitments from both nursing staff and trainers. Each track consisted of a planned seven sessions, comprising five two-hour sessions and two one-hour sessions. However, due to the COVID-19 pandemic and related national and organizational measures, sessions were canceled in four of the fourteen nursing home wards receiving the SELF-program: one fourth session, two fifth sessions, three sixth sessions, and four seventh sessions. This resulted in ten canceled sessions out of the originally planned 98. Consequently, the time investments from trainers and trainees on these specific wards amounted to five to eleven hours, rather than the standard twelve hours. The actor-led session had to be canceled in two out of the fourteen nursing home wards.

Considering that the trainees were trained to change daily routines, it was assumed that the impact of the training would be the same for both participating and non-participating clients. To calculate the intervention costs per client, the total costs were divided by the total of 192 clients residing in the wards for which the trained nursing staff were responsible.

#### Healthcare costs.

Healthcare costs were determined by multiplying the volumes of health services by their corresponding cost prices. The measured health services included interactions with the general practitioner, practice nurse, physiotherapist, dietician, occupational therapist, speech therapist, visits to outpatient clinics, hospital stays, ambulance rides, other modes of transportation to healthcare facilities, and medication usage. The cost prices utilized were sourced from the Dutch manual for costing in economic evaluations [22].

#### Other costs.

In addition to healthcare costs, the utilization of informal care was also evaluated. The unit price for informal care was derived from the manual mentioned earlier. However, productivity costs for clients, which are usually considered in economic evaluations from a societal perspective, were deemed irrelevant for this specific target population as the clients were not part of the labor force. Hence, these costs were excluded from measurement and analysis.

### Health outcomes

The primary outcome for the CEA was the degree of self-reliance in the daily functioning of geriatric clients, as measured by an adapted version of the GARS-4 [27, 28]. The questionnaire consists of four choice options per question, ranging from ‘without problems’ (score = 1) to ‘only with the assistance of others’ (score = 4). The standard GARS-4 questionnaire included the ADL short form, consisting of eleven questions. This resulted in a sum score ranging from 11, representing the most favorable score, to 44, representing the least favorable score. Additionally, the phrasing of the questions was slightly modified to make the version suitable for the first responsible caregiver of the clients.

In the CUA, the health outcome of interest was measured in quality-adjusted life years (QALYs), which were determined using the EuroQol-5D-5L (EQ-5D-5L), a generic health-related quality of life measurement instrument. The EQ-5D-5L assesses five dimensions of health-related quality of life: mobility, self-care, usual activities, pain/discomfort, and anxiety/depression. Each dimension is rated on a 5-point scale, ranging from ‘no problems’ (score = 1) to ‘extreme problems’ (score = 5). A Dutch tariff derived from the EQ-5D-5L was used [29]. The ratings on the five dimensions are then converted into utilities, which were multiplied by the timeframe to calculate QALYs for the CUA.

### Data preparation

All analyses were conducted based on the intention-to-treat principle, and the data were prepared for analysis following guidelines for handling missing data [30]. The missing data were found to meet the assumptions of missing at random, as there was no relationship between missing values and demographics, location, utilities, or healthcare consumption. Additionally, the dataset indicated that for each client and each measurement, the data were either entirely complete or entirely missing, allowing for imputation at an aggregated level, such as total costs per measurement. Missing data were imputed using predictive mean matching. The mean was drawn from five simulated datasets generated through the Markov Chain Monte Carlo method, with a maximum of ten iterations. It is assumed that at ten iterations the imputations have stabilized to a point where the order of variable imputation no longer affects the results [31]. The predictors in the model included demographic variables including age, gender, education level, marital status, care duration in the nursing home, baseline GARS-4 and utility scores, and baseline costs, and were limited to these to prevent overfitting of the model.

### Statistical analysis

The main analysis included a CEA and CUA from the societal perspective. We calculated costs of healthcare and non-healthcare costs in three steps: (1) assessment of the services and time consumed in the 3-month intervals between T0-T1 and T1-T2 (2) calculation of the associated costs in Euros, and (3) calculation of the incremental cost-effectiveness ratio (ICER) using the formula (C_i_–C_c_)/(E_i_–E_c_). Here C represents the sum of the average total costs per client during the 6 months between T0-T2, and E represents the mean difference in outcome at T2 in comparison with the number measured at T0 in the intervention (Ci and Ei) and the control (Cc and Ec) condition. For the cost-utility analysis (CUA), the QALY at follow-up was calculated by multiplying the patient’s utility score by 0.5 to represent the six-month follow-up period. A higher QALY was considered desirable. For the CEA, a lower GARS-4 score was deemed desirable. Therefore, for the CEA and calculation of the ICER, the effect needed to be inverted. In the case of death, the quality-adjusted life year (QALY) and costs were set at zero, and the GARS-4 sum score was set at 44, representing the least favorable score.

Stochastic uncertainty in the data was addressed using nonparametric bootstraps. Through the application of the bootstrapping technique, means were calculated, and 5000 ICERs were simulated. These ICERs were then plotted in cost-effectiveness planes, which visually represented the probability of the intervention being cost-effective compared to the control condition.

Additionally, cost-effectiveness acceptability curves (CEACs) were generated. The CEAC illustrates the probability of the intervention being considered cost-effective compared to CAU at various hypothetical WTP thresholds. It was determined that fixed WTP thresholds for the GARS-4 sum score and the QALY would not be used. There is no established fixed WTP threshold for the GARS-4 sum score. As for the QALY, the Dutch Healthcare Institute states that the threshold (ranging from €20,000 to €80,000 per QALY) should depend on the disease burden experienced by the participants [32]. In our study, this disease burden varied significantly.

### Sensitivity and subgroup analysis

Several sensitivity analyses were performed [33]. In the first analysis, ICERs were calculated based on costs and effects at the 3-month follow-up. This analysis aimed to assess any changes in cost-effectiveness and cost-utility between the 3- and 6-month follow-up periods. In the second analysis, a healthcare perspective was used, excluding informal care costs. This perspective aligns with the preferred approach in some countries and is not influenced by the informal care costs reported for clients in this trial. Furthermore, sensitivity analyses were conducted based on total cost figures, excluding intervention costs. This approach was chosen to reflect a scenario in which FFC-based routines are implemented as the default way of working, resulting in diluted per-client intervention costs. Lastly, an additional scenario analysis was conducted using data adjusted for baseline differences in outcomes. T-tests were performed to determine whether there were statistically significant differences in baseline outcome scores between the two conditions. Subgroup analyses were conducted for each of the three participating care organizations. The primary purpose of these subgroup analyses to investigate potential variations in the SELF-program’s cost-effectiveness across different organizational contexts.

### Ethics approval and consent to participate

The study was approved by the Medical Ethics Committee of the Zuyderland Hospital (METC-Zuyd; METCZ20210007). In line with Dutch regulation, no specific ethical approval was needed for this study according to the rules of the Medical Research Involving Human Subjects act (WMO). The study is registered in the International Clinical Trials Registry Platform: NL9189; https://trialsearch.who.int/Trial2.aspx?TrialID=NL9189. Consent to participate was obtained from nursing staff and geriatric clients. Nursing staff were given the choice whether or not to provide online informed consent to participate, and by confirming their choice with their online signature. For geriatric clients, written informed consent was obtained from either the client itself or a responsible party, generally a family member appointed as the primary contact person of the client.

## Results

### Baseline sample characteristics

Of the 246 clients who consented, 241 (98%) baseline questionnaires were completed by their primary responsible nursing caregiver. In total, 28 wards with a total of 241 clients were randomized (intervention, n = 115; control group, n = 126; Table 1). The wards were unevenly distributed among the three organizations because one care organization was larger than the others (15 wards vs. 7 wards vs. 6 wards). However, within each organization, wards were evenly distributed, with a 50−50 split between the intervention and control conditions. The mean age of the participants was 85.2 years (SD = 7.9) and most participants were female (75.9%). The majority either had a low (n = 132, 54.8%) or medium (n = 36, 14.9%) educational level, although for 63 clients the educational level was reported to be unknown. The mean GARS sum score at baseline was 29.8 (SD = 9.2), meaning geriatric clients could on average perform ADL independently but with great difficulty. The mean baseline GARS-4 sum scores were 28.4 (SD = 9.0) for intervention participants and 31.1 (SD = 9.2) for control participants and these showed to be significantly different at baseline (p = 0.022). The same goes for baseline EQ-5D-5L scores, which were 0.57 (SD = 0.24) for intervention participants and 0.45 (SD = 0.28) for control participants (p < 0.001).

**Table 1:** Baseline sample characteristics of geriatric clients.

	Total (N=241)	Intervention (N=115)	Control (N=126)
Organization 1:, n (%)	42 (17.4)	23 (20.0)	19 (15.1)
Organization 2:, n (%)	160 (66.4)	72 (62.6)	88 (69.8)
Organization 3:, n (%)	39 (16.2)	20 (17.4)	19 (15.1)
Age in years, mean (SD)	85.2 (7.9)	84.5 (7.8)	85.8 (8.0)
Gender, female, n (%)	183 (75.9)	92 (80.0)	91 (72.2)
Education level, n (%)			
Low	132 (54.8)	78 (67.8)	54 (42.8)
Medium	36 (14.9)	19 (16.5)	17 (13.5)
High	10 (4.1)	4 (3.5)	6 (4.8)
Unknown	63 (26.1)	14 (12.2)	49 (38.9)
Marital status, n (%)			
Married	42 (17.4)	11 (9.6)	31 (24.6)
In a relationship	5 (2.1)	3 (2.6)	2 (1.6)
Single/Divorced	39 (16.2)	26 (22.6)	13 (10.3)
Widow(er)	155 (64.3)	75 65.2)	80 (63.5)
Care duration in nursing home, years (SD)^a^	3.1 (2.7)	3.3 (3.2)	2.9 (2.3)
Informal Care, yes, n (%)	179 (74.3)	86 (74.8)	93 (73.8)
GARS-4 Baseline Sum Score, mean (SD)^b^	29.8 (9.2)	28.4 (9.0)	31.1 (9.2)
EQ-5D-5L Baseline Utility score, mean (SD)	0.51 (0.27)	0.57 (0.24)	0.45 (0.28)
Total costs in euro, mean (SD)	1,217 (4,168)	921 (2,636)	1,488 (5,183)

^a^SD = Standard deviation;

^b^Based on eleven items on ADL

**Fig 1:**
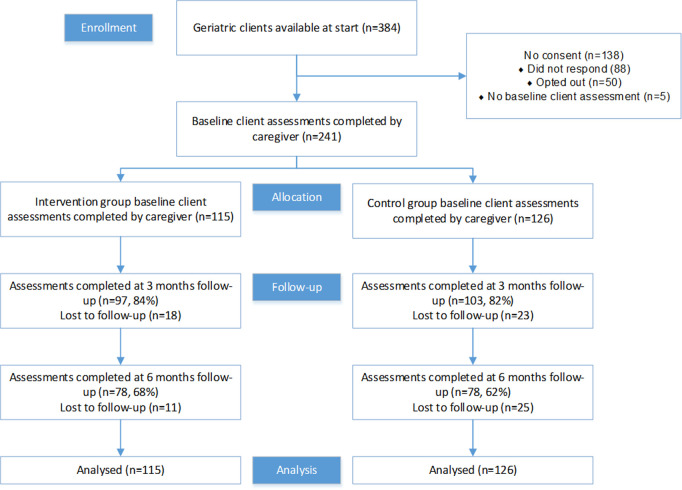
Consolidated Standards or Reporting Trials (CONSORT) diagram summarizing the progress of clients throughout the trial.

Loss to follow-up at 3 months was 16% (n = 18) for the intervention group and 18% (n = 23) for the control group. There was an additional loss to follow-up between 3 and 6 months, with n = 11 for the intervention group and n = 25 for the control group. As a result, there were 156 complete cases, with 78 in each condition. All analyses were performed according to the intention-to-treat principle, which involved imputing missing data and conducting analyses based on the sample of clients who had completed baseline client assessments (n = 241). See Fig 1 for the Consolidated Standards of Reporting Trials (CONSORT) flow diagram.

### Cost analysis

A total of 1,268 working hours were dedicated by trainers and trainees, averaging 14.41 hours per session. The opportunity costs for trainees were based on the hourly wages for nurse practitioners, as estimated by Statistics Netherlands [34]. These costs were calculated at €46.21 per hour per trainee, including holiday bonuses and indexed for 2022 prices. The costs for hiring actors amounted to €400 per session. Based on the number of clients who could potentially benefit from the intervention, the estimated total intervention costs per participant were calculated to be €371.52.

Mean total per-participant costs at baseline based on a 3-month recall period were €921.36 (SD = €2,636.11) for the intervention and €1,487.72 (SD = €5,182.89) for the control participants Table 2.

**Table 2:** Mean per-participant costs by condition at baseline based on a 3-month recall period (€, indexed for 2022). Data are presented as mean (standard deviation). The cost prices utilized were sourced from the Dutch manual for costing in economic evaluations [22].

	Cost per unit	Intervention (N = 115)	Control (N = 126)
Total healthcare costs			
General practitioner	€40.23/consultation	€141 (€168)	€146 (€133)
Practice nurse	€20.73/consultation	€3 (€15)	€6 (€29)
Physiotherapist	€40.23/consultation	€111 (€197)	€151 (€233)
Dietician	€40.23/consultation	€15 (€35)	€23 (€39)
Occupational therapist	€40.23/consultation	€12 (€33)	€53 (€97)
Speech therapist	€36.58/consultation	€4 (€173)	€8 (€26)
Outpatient clinics	€110.95/visit	€16 (€49)	€42 (€114)
Hospital stays	€580.33/night	€50 (€349)	€534 (€4.755)
Ambulance rides	€627.88/ride	€22 (€116)	€10 (€79)
Other costs			
Informal care costs	€17.07/hour	€548 (€2,561)	€515 (€1,264)
Total costs		€921 (€2,636)	€1,488(€5,183)

During 6 months follow-up, from a societal perspective, total costs were lower in the intervention vs control group (mean, €1,517[SD, €2266] and €2,101 [SD, €8224], respectively). From a healthcare perspective, however, costs were higher for the intervention vs the control group (mean, €1,163 [SD, €1792] and €607 [SD, €856], respectively). This means that the inclusion or exclusion of informal care costs in the analyses had a significant impact on the results of the CEA and CUA.

### Cost-effectiveness analysis

**Fig 2:**
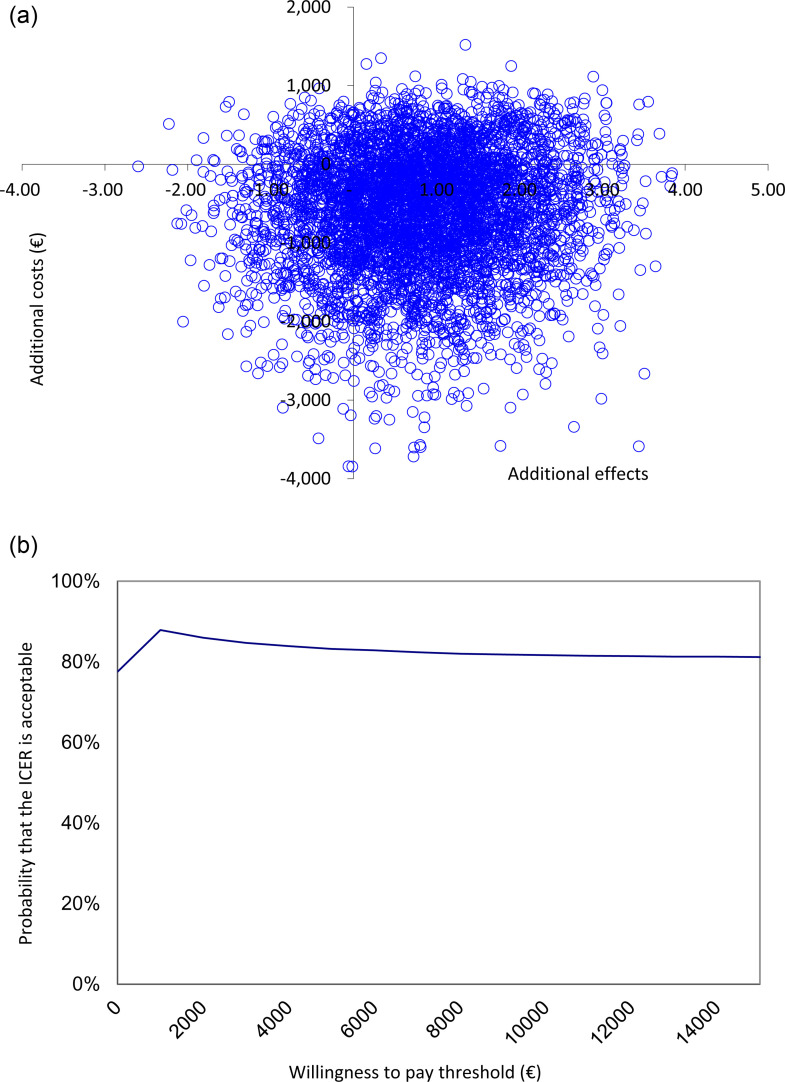
A. Cost-effectiveness plane for the base case with GARS-4 as outcome measure. B. Cost-effectiveness acceptability curve for the base case with GARS-4 as outcome measure.

Table 3 (upper panel) presents the results for the main cost-effectiveness analysis. From a societal perspective, the total costs at 6 months follow-up were lower in the intervention condition as compared to the control condition. The incremental costs, representing the difference in average costs per participant between the intervention and control condition, amounted to -€584. The incremental effects observed were a 0.81 point decrease on the GARS-4 sum score. This means that the intervention dominated CAU. The distribution of bootstrapped ICERs and the CEACs are presented in Figs 2A and 2B. The probability that the intervention is cost-effective compared to care as usual at a WTP of €0/point reduction on the GARS-4 sum score is 61% and reaches a maximum of 88% at a WTP threshold of €1,000.

**Table 3:** Summary statistics for the main, sensitivity, and subgroup cost-effectiveness bootstrap analyses.

Condition	Costs, €^a^	Effect^b^	ICER^c^	North East	North West (inferior)	South West	South East (dominant)
*Main analysis (6 months follow-up)*							
Control (n = 126)	2,101 (540–3713)	−5.25 (−6.89 to −4.19)					
Intervention (n = 115)	1,517 (1,098–1,935)	−4.44 (−5.92 to −2,95)	-€268 (Dominant)	19%	4%	17%	61%
*Analysis from a healthcare perspective (6 months follow-up)*							
Control (n = 126)	607 (456–758)	−5.25 (−6.89 to −4.19)					
Intervention (n = 115)	1,163 (832–1,494)	−4.44 (−5.92 to −2,95)	€410	79%	21%	0%	0%
*Analysis at 3 months follow-up*							
Control (n = 126)	1,586 (145–3027)	−3.04 (−4.09 to −1.99)					
Intervention (n = 115)	1,030 (734–1,326)	−1.78 (−2.95 to −0.61)	-€312 (Dominant)	24%	1%	4%	71%
*Excluding intervention costs (6 months follow-up)*							
Control (n = 126)	2,101 (540–3713)	−5.25 (−6.89 to −4.19)					
Intervention (n = 115)	1,145 (727–1,564)	−4.44 (−5.92 to −2,95)	-€491 (Dominant)	8%	1%	20%	71%
*Adjusted for baseline differences (6 months follow-up)*							
Control (n = 126)	2,101 (540–3713)	−6.08 (−7.43 to −4.73)					
Intervention (n = 115)	1,517 (1,098–1,935)	−4.44 (−5.92 to −2,95)	−268 (Dominant)	22%	1%	4%	73%
*Subgroup analysis: Organization 1 (6 months follow-up)*							
Control (n = 19)	635 (410–860)	−3.68 (−6.41 to −0.96)					
Intervention (n = 23)	1,618 (359–2,877)	−1.55 (−5.16 to 2.07)	€306	83%	16%	0%	1%
*Subgroup analysis: Organization 2 (6 months follow-up)*							
Control (n = 88)	1,844 (566–3,122)	−4.73 (−6.19 to −3.27)					
Intervention (n = 72)	1,399(863–1,935)	−4.64 (−6.59 to −2.69)	€51	18%	11%	35%	36%
*Subgroup analysis: Organization 3 (6 months follow-up)*							
Control (n = 19)	4,541 (−3,526–12,609)	−8.89 (−14.01 to −3.76)					
Intervention (n = 20)	1,723 (1,153–2,294)	−8.11 (−11.9 to −4.3)	-€231(Dominant)	21%	15%	26%	38%

^a^Mean costs (95% Confidence Intervals) per participant at 2022 prices;

^b^Mean effects (95% Confidence Intervals) presented are points reduction on the GARS-4 between baseline and follow-up;

^c^The presented ICER is the 50thpercentile of 5,000 bootstrap replications of the ICER.

From thehealthcare perspective, the total costs at 6 months follow-up were higher in the intervention condition as compared to the control condition. The incremental costs amounted to €556. The bootstrapped median ICER was €410 per point reduction on the GARS-4. The distribution of bootstrapped ICERs and the CEACs are presented in Supporting information (S1 Fig). The probability that the ICER is acceptable at a WTP of €0/point reduction on the GARS-4 sum score is 0% and reaches a ceiling of 76% at a WTP threshold of €5,000.

### Cost-utility analysis

**Fig 3:**
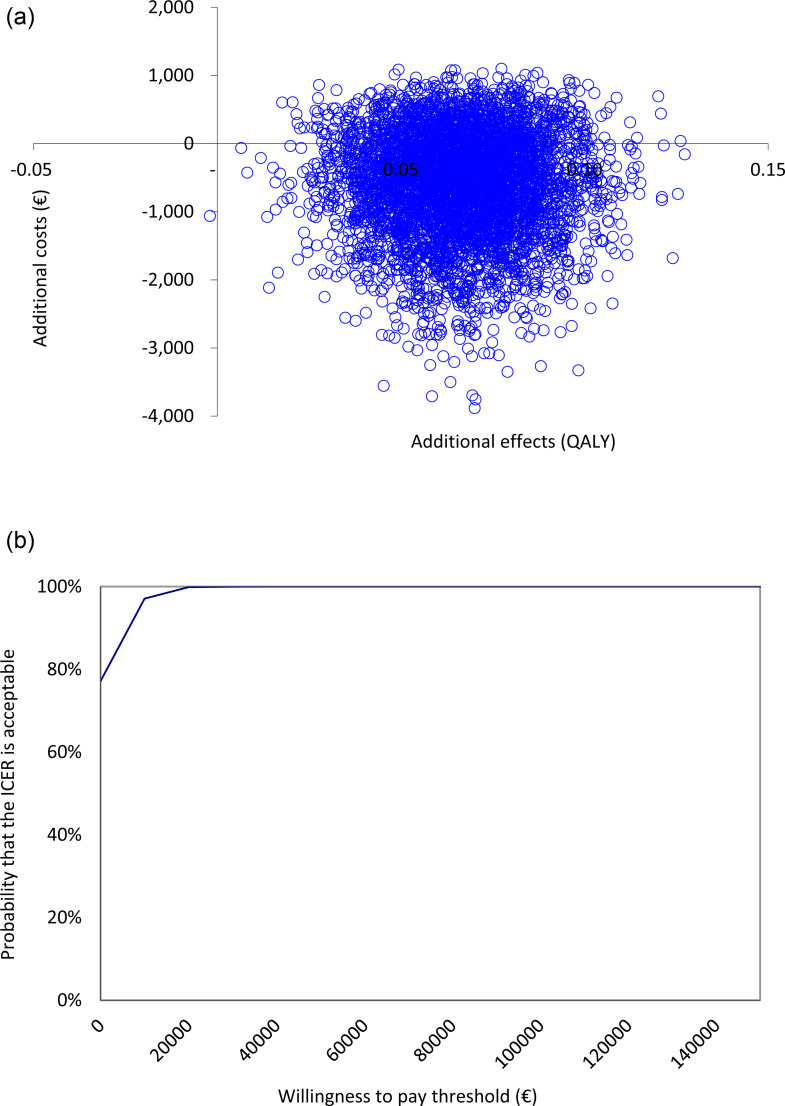
A. Cost-effectiveness plane for the base case with QALY as outcome measure. B. Cost-effectiveness acceptability curve for the base case with QALY as outcome measure.

Table 4 (upper panel) presents the results for the main cost-utility analysis. The incremental effects observed were an increase 0.07 QALYs at 6 months follow-up. This means that, also for the main cost-utility analysis, the intervention dominated CAU. The distribution of bootstrapped ICERs and the CEACs are presented in Figs 3A and 3B. The probability that the ICER is acceptable at a WTP of €0/QALY is 77% and reaches 100% at WTP thresholds of €16,000 and higher.

**Table 4:** Summary statistics for the main, sensitivity, and subgroup cost-utility bootstrap analyses.

Condition	Costs, €^a^	Effect^b^	ICER^c^	North East	North West (inferior)	South West	South East (dominant)
*Main analysis (6 months follow-up)*							
Control (n = 126)	2,101 (540–3713)	0.17 (0.15 to 0.20)					
Intervention (n = 115)	1,517 (1,098–1,935)	0.24 (0.22 to 0.26)	-€7,910 (Dominant)	23%	0%	0%	77%
*Analysis from a healthcare perspective (6 months follow-up)*							
Control (n = 126)	607 (456–758)	0.17 (0.15 to 0.20)					
Intervention (n = 115)	1,163 (832–1,494)	0.24 (0.22 to 0.26)	€8,356	100%	0%	0%	0%
*Analysis at 3 months follow-up*							
Control (n = 126)	1,586 (145–3027)	0.11 (0.10 to 0.12)					
Intervention (n = 115)	1,030 (734–1,326)	0.14 (0.13 to 0.15)	-€15,429 (Dominant)	23%	0%	0%	77%
*Excluding intervention costs (6 months follow-up)*							
Control (n = 126)	2,101 (540–3713)	0.17 (0.15 to 0.20)					
Intervention (n = 115)	1,145 (727–1,564)	0.24 (0.22 to 0.26)	-€13,378 (Dominant)	9%	0%	0%	91%
*Adjusted for baseline differences (6 months follow-up)*							
Control (n = 126)	2,101 (540–3,713)	0.22 (0.19 to 0.25)					
Intervention (n = 115)	1,517 (1,098–1,935)	0.24 (0.22 to 0.26)	−16,757 (Dominant)	20%	3%	11%	66%
*Subgroup analysis: Organization 1 (6 months follow-up)*							
Control (n = 19)	635 (410–860)	0.18 (0.11 to 0.25)					
Intervention (n = 23)	1,618 (359–2,877)	0.25 (0.19 to 0.31)	€11,350	94%	5%	0%	1%
*Subgroup analysis: Organization 2 (6 months follow-up)*							
Control (n = 88)	1,844 (566–3,122)	0.18 (0.15 to 0.20)					
Intervention (n = 72)	1,399 (863–1,935)	0.24 (0.21 to 0.27)	-€6,095 (Dominant)	29%	0%	0%	71%
*Subgroup analysis: Organization 3 (6 months follow-up)*							
Control (n = 19)	4,541 (−3,526–12,609)	0.14 (0.09 to 0.19)					
Intervention (n = 20)	1,723 (1,153–2,294)	0.21 (0.17 to (0.27)	-€34,256 (Dominant)	36%	1%	1%	63%

^a^Mean costs (95% Confidence Intervals) per participant at 2022 prices;

^b^Mean effects (95% Confidence Intervals) presented are the QALYs gained between baseline and follow-up;

^c^The presented ICER is the 50thpercentile of 5,000 bootstrap replications of the ICER.

From the healthcare perspective, the bootstrapped median ICER was €8,356 per QALY. The distribution of bootstrapped ICERs and the CEACs are presented in Supporting information (S2 Fig). The probability that the ICER is acceptable at a WTP of €0/QALY is 0% and reaches 100% at a WTP threshold of €30,000.

### Sensitivity and subgroup analyses

The results of the scenario and sensitivity analyses—including the 3-month follow-up analysis, the analysis excluding intervention costs, and the scenario analyses adjusted for baseline differences in the GARS and EQ-5D—confirm the robustness of the main analyses (Tables 3 and 4). Similar to the main analysis, the ICERs indicate that the intervention dominates CAU. The distributions of bootstrapped ICERs and the CEACs are provided in Supporting information (S3 Fig). The subgroup analyses yielded mixed and highly uncertain results. In terms of the GARS-4 outcome measure, the intervention outperformed CAU condition in one of the three organizations, while the probability of cost-effectiveness increased with higher WTP thresholds across all three organizations. The same trend was observed for the QALY outcome measure. The distributions of bootstrapped ICERs and the CEACs for the subgroups are presented in Supporting information (S4 Fig).

## Discussion

### Summary

This multicenter cluster-randomized trial aimed to assess the cost-effectiveness and cost-utility of the SELF-program compared to CAU. SELF demonstrated a reduction in the GARS-4 sum score and an increase in QALYs over a 3-month and 6-month follow-up period. From a societal perspective, average costs were lower in the intervention condition, while from a healthcare perspective, they were higher. This indicates that the intervention dominated CAU in terms of both CEA and CUA from a societal perspective, but showed positive ICERs from a healthcare perspective. Notably, the probability of the ICER being considered acceptable increased with higher WTP thresholds for both outcome measures. The discrepancies between the perspectives highlight the significant influence of informal care costs on the results of the economic evaluation.

### Comparison with existing literature

The number of studies evaluating the cost-effectiveness of FFC-based or reablement interventions is limited. There have not been assessments of inpatient interventions directly comparable to the intervention of the SELF study in terms of cost-effectiveness and cost-utility in the available literature. Only studies evaluating outpatient care interventions are present, and their results are mixed. In a UK study, individuals receiving home care reablement were compared with a group receiving conventional home care services. At a £30,000 threshold for each enhancement in health-related quality of life, the probability of cost-effectiveness of the intervention compared to control was high (99%) when considering both health and social care costs, and nearly certain (just under 100%) when considering only social care costs. Even at a more stringent threshold of £20,000 per health-related outcome gain, the probability of cost-effectiveness remained very high, at 98% for both health and social care costs, and 99% for social care costs alone [18]. Similarly encouraging findings were reported in a study by Kjerstad & Tuntland in Norway, where, compared to standard care, reablement proved to be a more cost-effective option. The reablement group demonstrated significantly higher levels of performance and satisfaction in daily activities compared to the control group, all achieved at a lower cost [19]. On the contrary, a study in the Netherlands demonstrated that an reablement training program for homecare staff did not improve outcomes or reduce costs and was not cost-effective from a societal perspective compared to usual care in Dutch older adults receiving homecare [20]. The findings from our recent study suggest that FFC-based interventions may still be superior when compared to usual care. Future research could potentially reveal differences in the level of cost-effectiveness between inpatient and outpatient FFC-based interventions.

### Strengths and limitations

A major strength of this study is the integration of economic data collection within the RCT design, and the implementation of the SELF-program in real-life conditions, thereby enhancing the reliability and robustness of the results. The study also utilized both a quality of life instrument and an ADL instrument sensitive to measuring the capabilities of nursing home clients, thereby increasing the likelihood of detecting any impacts resulting from the intervention.

There are several limitations to consider in the analyses. First, the SELF-program training was conducted during the first three months of resident follow-up. By training nursing staff early in the follow-up period, we aimed to assess the immediate, real-world impact of the SELF-program as it would likely be implemented in practice. This concurrent training and implementation period may have contributed to observed cost and outcome effects, as nursing staff were actively adapting new skills during this time.

Second, data for the economic evaluation of the SELF-program was collected from the primary caregivers responsible for nursing home clients and these caregivers knew whether they received the intervention. Clients with somatic complaints and even with mild psychogeriatric complaints may be capable of estimating their level of self-reliance in ADL. However, to address uniformity in data collection, all client data were collected by the primary responsible caregiver, independent of the clients somatic or psychogeriatric background. While this choice can be justified from both a pragmatic and academic standpoint, there is a potential risk of bias, including recall bias, as the primary caregivers may not have had complete and up-to-date information on the healthcare utilization of the study participants.

Third, the participant group was heterogeneous in terms of age, duration of care in the nursing home, health-related quality of life, and capabilities. The indirect design of the study, where the intervention targeted nursing staff while costs and outcomes were measured for clients, along with the goal of assessing cost-effectiveness and cost-utility for all affected participants, required a less restrictive approach to inclusion criteria. However, given the broad inclusion criteria, the sample size might have been too low and this may limit the transferability of the results to other care organizations.

Fourth, in the Netherlands, the WTP threshold typically falls between €20,000 and €80,000 per QALY, with the specific range depending on the disease burden of the target population [32]. Given the significant variation in disease burden among the participants in our study, a fixed WTP threshold could not be established.

Fifth, conducting subgroup analyses at the organizational level was challenging due to the relatively low number of participants in two out of the three organizations. Bootstrap analyses revealed a higher distribution of simulated ICERs for these two organizations compared to the organization with a larger subsample. The level of uncertainty associated with these organizations cannot be adequately addressed through analysis techniques, thus limiting the generalizability of the results within the participating organizations.

Finally, we did not investigate the factors driving differences in informal care costs between the two arms. Detailed data on the medical and personal factors influencing both healthcare and informal care costs were not collected, which limits our ability to identify potential underlying causes for these variations.

## Conclusion

The SELF-program is likely to offer good value for money and provides suggestive evidence for the continued development of FFC-based approaches in providing care for nursing home clients. There is a need for refinement and additional testing of the approach to ascertain whether the magnitude and scope of cost savings and improvements in outcomes can be reproduced. It remains uncertain whether the results observed under controlled trial conditions will be replicated under routine service delivery conditions.

## Supporting information

S1 Fig Cost-effectiveness plane and cost-effectiveness acceptability curve with GARS-4 as the outcome measure at 6-months follow-up from a healthcare perspective. (DOCX)

S2 Fig Cost-effectiveness plane and cost-effectiveness acceptability curve with QALY as the outcome measure at 6-months follow-up from a healthcare perspective. (DOCX)

S3 Fig Cost-effectiveness planes and cost-effectiveness acceptability curves for the scenario and sensitivity analyses. (DOCX)

S4 Fig Cost-effectiveness planes and cost-effectiveness acceptability curves for the subgroup analyses. (DOCX)

S1 Table Consolidated Health Economic Evaluation Reporting Standards 2022. (DOCX)

S2 Table Unit prices used for intervention and healthcare costs calculations. (DOCX)

S1 File Minimal anonymised dataset SELF study - RAW data - English. (SAV)
