# Supplementary material for: A multi-center trial-based economic evaluation of the SELF-program: A function-focused care program for nursing staff providing long-term care for geriatric clients in nursing homes compared to care as usual
Source: PLoS One. 2025 Jul 2;20(7):e0320649. doi: 10.1371/journal.pone.0320649 (PMC12221074; doi:10.1371/journal.pone.0320649)
Supplement: S1 Fig — (DOCX) [file pone.0320649.s001.docx]

S1a Fig. Cost-effectiveness plane with GARS-4 as the outcome measure at 6-months follow-up from a healthcare perspective

S1b Fig. Cost-effectiveness acceptability curve with GARS-4 as the outcome measure at 6-months follow-up from a healthcare perspective
