# Supplementary material for: A multi-center trial-based economic evaluation of the SELF-program: A function-focused care program for nursing staff providing long-term care for geriatric clients in nursing homes compared to care as usual
Source: PLoS One. 2025 Jul 2;20(7):e0320649. doi: 10.1371/journal.pone.0320649 (PMC12221074; doi:10.1371/journal.pone.0320649)
Supplement: S3 Fig — (DOCX) [file pone.0320649.s003.docx]

S3a Fig. Cost-effectiveness plane with GARS-4 as the outcome measure at 3-months follow-up

S3b Fig. Cost-effectiveness acceptability curve with GARS-4 as the outcome measure at 3-months follow-up

S3c Fig. Cost-effectiveness plane with QALY as the outcome measure at 3-months follow-up

S3d Fig. Cost-effectiveness acceptability curve with QALY as the outcome measure at 3-months follow-up

S3e Fig. Cost-effectiveness plane with GARS-4 as the outcome measure at 6-months follow-up excluding intervention costs

S3f Fig. Cost-effectiveness acceptability curve with GARS-4 as the outcome measure at 6-months follow-up excluding intervention costs

S3g Fig. Cost-effectiveness plane with QALY as the outcome measure at 6-months follow-up excluding intervention costs

S3h Fig. Cost-effectiveness acceptability curve with QALY as the outcome measure at 6-months follow-up excluding intervention costs

S3i Fig. Cost-effectiveness plane with GARS-4 as the outcome measure at 6-months follow-up adjusted for baseline differences

S3j Fig. Cost-effectiveness acceptability curve with GARS-4 as the outcome measure at 6-months adjusted for baseline differences

S3k Fig. Cost-effectiveness plane with QALY as the outcome measure at 6-months follow-up adjusted for baseline differences

S3l Fig. Cost-effectiveness acceptability curve with QALY as the outcome measure at 6-months adjusted for baseline differences
