# Supplementary material for: A multi-center trial-based economic evaluation of the SELF-program: A function-focused care program for nursing staff providing long-term care for geriatric clients in nursing homes compared to care as usual
Source: PLoS One. 2025 Jul 2;20(7):e0320649. doi: 10.1371/journal.pone.0320649 (PMC12221074; doi:10.1371/journal.pone.0320649)
Supplement: S2 Table — (DOCX) [file pone.0320649.s006.docx]

S2 Table. Unit prices used for intervention and healthcare costs calculations

| **Item** | **Source** | **Year** | **Unit** | **Unit Price (€)** | **Unit price, indexed for 2022 (€)** |
| --- | --- | --- | --- | --- | --- |
| ***Intervention unit prices*** |  |  |  |  |  |
| Actor | Study | 2022 | Per session | 400 | 400,00 |
| Participants | Statistics Netherlands 2022 | 2020 | Per hour | 40,91 | 46,21 |
| ***Health services unit prices*** |  |  |  |  |  |
| General practitioner | Hakkaart et al. 2015 | 2014 | Per contact | 33 | 40,23 |
| Practice nurse | Hakkaart et al. 2015 | 2014 | Per contact | 17 | 20,73 |
| Physiotherapist | Hakkaart et al. 2015 | 2014 | Per contact | 33 | 40,23 |
| Dietician | Based on physiotherapist | 2014 | Per contact | 33 | 40,23 |
| Occupational therapist | Hakkaart et al. 2015 | 2014 | Per contact | 33 | 40,23 |
| Speech therapist | Hakkaart et al. 2015 | 2014 | Per contact | 30 | 36,58 |
| Outpatient clinics | Hakkaart et al. 2015 | 2014 | Per visit | 91 | 110,95 |
| Hospital stays | Hakkaart et al. 2015 | 2014 | Per day | 476 | 580,33 |
| Ambulance rides | Hakkaart et al. 2015 | 2014 | Per ride | 515 | 627,88 |
| Other forms of transportation | Hakkaart et al. 2015 | 2014 | Per ride | 272 | 331,62 |
| Informal care | Hakkaart et al. 2015 | 2014 | Per hour | 14 | 17,07 |
